# Effect of Interaction between Early Menarche and Genetic Polymorphisms on Triglyceride

**DOI:** 10.1155/2019/9148920

**Published:** 2019-02-25

**Authors:** Ho-Sun Lee, Sangseob Leem, Bermseok Oh, Taesung Park

**Affiliations:** ^1^Interdisciplinary Program in Bioinformatics and Department of Statistics, Seoul National University, 1 Kwanak-Ro, Kwanak-gu, Seoul 151-747, Republic of Korea; ^2^Department of Biochemistry and Molecular Biology, School of Medicine, Kyung Hee University, Seoul 02447, Republic of Korea

## Abstract

Early menarche has been associated with increased risk of metabolic syndrome. Therefore, investigating the association of each component of metabolic syndrome with age at menarche, and interactions between them, might lead to a better understanding of metabolic syndrome pathogenesis. In this study, we evaluated age at menarche for risk of metabolic syndrome and associations with its components. As a result, the risk of MetS incidence was significantly increased only at ≤12 years of age at menarche (OR = 1.91, *P* < 0.05). Women with early menarche (≤12 years) had significantly higher levels of triglycerides (*β* coefficient = 37.83, *P* = 0.02). In addition, hypertriglyceridemia was significantly increased at early menarche with 1.99 (95% CI: 1.16–3.41, *P* < 0.01). With GWAS-based pathway analysis, we found the type 2 diabetes mellitus, stress-activated protein kinase signaling, and Jun amino-terminal kinase cascade pathways (all nominal *P* < 0.001, all FDR < 0.05) to be significantly involved with early menarche on triglyceride levels. These findings may help us understand the role of early menarche on triglyceride and interaction between gene and early menarche on triglyceride for the development of metabolic syndrome.

## 1. Introduction

Menarche, defined as the first menstrual period in a woman's life, is a marker of female puberty and the onset of ovarian and other endocrine functions related to reproductive capacity. Age of menarche has declined over the last several decades through improvement of socioeconomic conditions or exposure to environmental chemicals and received a great deal of attention as having important health implications [1]. Age at menarche (AAM) can be a risk factor for disease, and there were several studies on the interaction between AAM and genetic factors especially for breast cancer susceptibility [2–4]. Early menarche is often defined as menarche before the age of 12 years (≤11 years old), but some investigators base definition on menarche at ≤12 years [5]. A number of studies have reported associations between early menarche onset and intermediate quantitative traits such as increased blood glucose levels [6], impaired glucose tolerance [7], and insulin resistance [8], further supporting a link between early pubertal development and an adverse metabolic profile. Others have reported adverse metabolic consequences (e.g., diabetes) arising from early sexual maturation [9, 10]. One possible mechanism for this association is a direct link between puberty timing and glucose regulation, supported by the insulin-sensitizing agent metformin delay of menarche in girls with precocious puberty [11]. It is conceivable that part of the association between menarche onset and type 2 diabetes risk is explained by increased adiposity [9, 10]. However, these data are not consistent, and the relationship between AAM and metabolic components remains poorly understood. Metabolic syndrome (MetS) is considered a worldwide epidemic. In general, the International Diabetes Federation estimates that one-quarter of the world's adult population has MetS [12]. Higher socioeconomic status, sedentary lifestyle, and high body mass index (BMI) significantly associate with MetS. Data from the National Health and Nutrition Examination Survey (NHANES, 1999–2006) reported that nearly 34% of all U.S. adults, and 50% of those aged 60 years or older, were estimated to have MetS [12]. The prevalence of MetS in Korea has also been steadily increasing in recent years, from 24.9%, in 1998, to 31.3%, in 2007; this prevalence is relatively high compared to those of other Asian countries [13]. MetS is characterized by the clustering of five pathological traits, including large waist circumference, hypertriglyceridemia, low-density lipoprotein (LDL) cholesterol level, hypertension, and hyperglycemia. However, mechanisms and differences, correlated with the relative risk of susceptibility to MetS, remain poorly characterized.

To date, genome-wide association studies (GWAS) have identified several susceptibility regions and genes for MetS and its component phenotypes [14, 15]. Since the first report from a GWA study of obesity, an increasing number of genetic factors have been shown to associate with several traits of obesity and MetS. Despite the success of recent GWAS, the identified variants explain only a small proportion of the heritability of most complex diseases [16]. Heritability estimates of MetS range from approximately 10 to 30% [17] among individuals; therefore, implicated genes may interact synergistically with environmental factors, in the pathogenesis of MetS.

Here, we investigated the role of AAM on MetS components for MetS risk. Specifically, we examined the association between AAM and levels of individual component of MetS such as triglyceride (TG). Furthermore, we evaluated GWAS-identified genetic variants for interaction effects with MetS components using a pathway-based analysis and characterized the role of AAM in the pathogenesis of MetS.

## 2. Methods

### 2.1. Study Design and Participants

This study, an ongoing prospective investigation, is a population-based cohort included in the Korean Genome and Epidemiology Study (KoGES) [18]. Originally, 5018 woman participants, aged 40 to 69 years, were recruited from the Korean rural (Ansung) and urban (Ansan) communities at baseline, as part of the Korean Association Resource Project (KARE). From 2007 to 2008, the samples were scrutinized for quality control purposes, and 3493 participants remained. We excluded those with reported AAM of ≤10 years (*n* = 3) or ≥19 years (*n* = 272) or missing (*n* = 41), probably due to recall error or the presence of a comorbid pathological condition. Finally, information was available from 3180 women for the study of AAM.

These cohorts included a standardized health interview, using well-established questions, to determine the demographic and socioeconomic characteristics of all subjects, including menstruation history. AAM was defined as the age of the first menstrual period. Women reported their AAM in single years and were classified for this study as having an AAM of ≤12, 13, 14, 15, 16, or ≥17 years [19]. Comprehensive health examinations, including evaluation of anthropometric indexes, measurement of blood pressure, questionnaire-based interviews, and collection of biospecimens for assays (e.g., C-reactive protein), were conducted biennially by health professionals who were trained with a standardized protocol. Women who smoked regularly during the previous 12 months were classified as current smokers. Levels of physical activity were semiquantitatively assessed using a questionnaire previously validated against objective methods [20] and were coded as inactive, moderately inactive, moderately active, and active. Educational attainment was categorized into three groups: less than 7 years (elementary school graduates), 7–9 years (middle school graduates), and more than 10 years (high school graduates). Monthly household income was also categorized into three groups: less than $1000 USD (in 2014), $1000–2000, and ≥$2000 [21]. In addition, we performed the additional analysis for oxidative stress-related exposure with OBS score and inflammatory marker with C-reactive protein (CRP) at each AAM [22, 23]. Written informed consent was obtained from all participants at the KoGES. This study was approved by the Institutional Review Board of Seoul National University.

### 2.2. Definition of the Metabolic Syndrome and Its Components

We used the definition of MetS proposed by the criteria of the National Cholesterol Education Program Adult Treatment Panel III (NCEP-ATP III) guidelines [24]. As detailed in our study, participants having three or more of the following criteria were defined as having MetS, except for the determination of central obesity. Waist circumference cut-off values were used based on guidelines from the Korean Society for the Study of Obesity [25]: (1) central obesity, given as waist high circumference (≥85 cm); (2) high concentration of serum triglycerides (≥150 mg/dl); (3) low concentrations of serum HDL cholesterol (<50 mg/dl); (4) hypertension (systolic/diastolic pressure ≥ 130/85 mmHg) or antihypertensive medication; and (5) high concentrations of fasting glucose (≥100 mg/dl) or antidiabetic medication.

### 2.3. Genotyping

The data used for GWAS was obtained from KoGES, which was conducted by the Korean National Institute of Health. DNA samples were isolated from the peripheral blood of all participants and genotyped, using the Affymetrix Genome-Wide Human SNP array 5.0 (Affymetrix Inc., Rockville, MD, USA). Quality control procedures were adopted, such as missing genotype frequency > 0.5% and minor allele frequency (MAF) ≤ 0.05. After sample and genotype quality controls, 344,396 SNPs for 3180 individuals were available in the KARE database [26, 27]. The KARE data used to support the findings of this study are restricted by the Institutional Review Board of the Korean National Institute of Health, who can contact at National Biobank of Korea (http://koreabiobank.re.kr, 82–1661-9070).

### 2.4. Statistical Analysis

For demographics and characteristics of subjects, data were presented as means and standard deviations for continuous variables, or a percentage (%), for categorical variables (Table 1). Differences among subjects in different groups were detected using the Kruskal-Wallis test, for continuous variables, and the chi-square test, for categorical variables.

**Figure 1:**
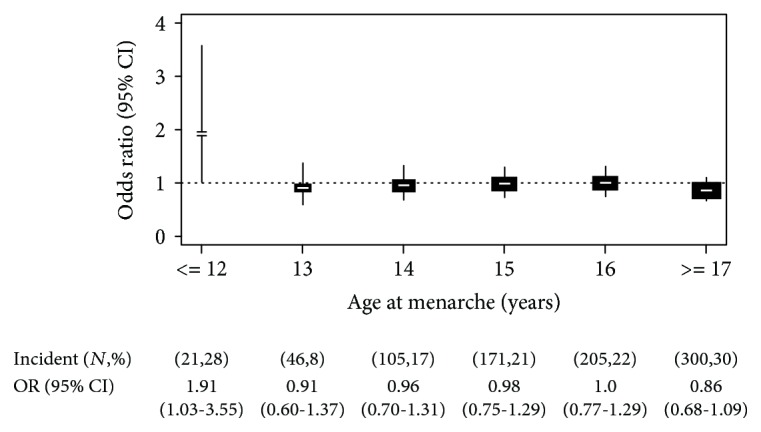
Risk of MetS in each age of menarche. Odds ratio (OR) and 95% confidence interval (CI) of Mets by each age at menarche. *N* is the number of incidence in each age of menarche; % is the percent of incidence in each age of menarche. The size of rectangle represents the number of samples. The vertical line indicates the overall OR on each age of menarche. ORs were adjusted for area, age, income, education, and C-reactive protein levels. The reference category was menarche at 16 years of age.

**Figure 2:**
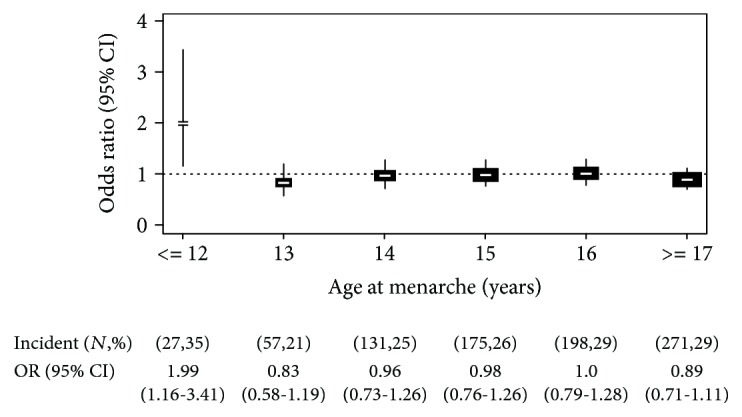
Risk of hypertriglyceridemia in each age at menarche. Odds ratio (OR) and 95% confidence interval (CI) of hypertriglyceridemia by each age at menarche. *N* is the number of incidence in each age of menarche; % is the percent of incidence in each age of menarche. The size of rectangle represents the number of samples. The vertical line indicates the overall OR on each age of menarche. ORs were adjusted for area, age, income, education, and C-reactive protein levels. The reference category was menarche at 16 years of age.

At first, logistic regression analyses were performed to determine the odds ratios (ORs) of MetS (Figure 1), and its five components, depending on each year of menarche, with 16 years of age as a reference group (Figure 2). Logistic regression models were adjusted for age, area, income, education, and CRP levels. Next, we evaluated beta coefficients for each year of menarche, using linear regression analyses for individual components of MetS, with 16 years of age as a reference group (Table 2). This reference group was chosen because it covers the median of AAM (median age = 16.0) [28].

To test the interactions between SNPs and AAM, we performed interaction tests for identifying genetic variants associated with MetS and its characteristic components. In addition, we performed the joint test of the main and interaction effects with one degree of freedom (df). We then used the following equation:

(1) $$\begin{array}{c} Y_{i}\left(\text{or log it }p_{i}\right)=\beta_{0}+\beta_{1}E_{i}+\beta_{\text{main}}G_{\text{i}}+\beta_{\text{int}}{GE}_{\text{i}}, \end{array}$$

where *Y*_*i*_ is the MetS component for individual *i* and *p*_*i*_ = *P*_*r*_ (*Y*_*i*_=1) of MetS for a binary trait. We then used the Wald test statistic to report the results of the joint (*P*_joint_) test, with two dfs and the gene-risk factor (*P*_int_) with one df, referring to their respective null hypotheses of the joint effects (*β*_main_ = 0 and *β*_int_ = 0) and of the interaction effect (*β*_int_ = 0), respectively.

The critical *P* values for accessing the significance of interaction were calculated by Bonferroni correction (*P* < 1.45 × 10^−7^) or the false discovery rate (FDR), with a *q* value less than 0.05. Quantile-quantile (QQ) plots of the *P* values for the joint test and interaction analysis suggested a moderate inflation of the genome-wide analysis only for TG. The inflation factors were 1.32 and 1.24 for interaction and joint effects, respectively, for TG in KARE dataset (Supplemental Figure S2). There were no genomic inflations for other components (*λ* < 1.05). Therefore, for genomic controls of TG, the inverse normal transformed TG was used as a response, adjusting for age, area, income, education, and CRP levels for KARE. All analyses were performed using the R software version 2.11.1 (http://www.r-project.org/). Data management, descriptive statistics for the covariates and outcome variates, and the regression analyses were conducted using the *Stats* R package.

### 2.5. Pathway-Based Analysis

Pathway-based approaches using GWAS data are now used routinely to study complex diseases [29]. To analyze pathways interacting AAM with TG genetic polymorphism, we used the improved gene set enrichment analysis (GSEA) [29]. This approach has the advantage that genetic variant associations, mapping to any genes, provide insight into biological functions, signal pathways, and mechanisms of disease.

In our study, 344,396 SNPs were mapped to genes within 20 kb boundaries. Pathways consisting of <20 or >200 genes were excluded from further analysis, to reduce the multiple testing issue and avoid testing overly narrow or broad functional categories [22, 27]. A false discovery rate (FDR) was used for multiple testing correction, with *q* values < 0.05 considered significant. Improved GSEA approaches use a comprehensive pathway and gene set database from SNP data. Pathway-related information is obtained from the KEGG (Kyoto Encyclopedia of Genes and Genomes pathway database), BioCarta, and GO (gene ontology) databases [27].

## 3. Results

### 3.1. Characteristics of Participants

A total of 3180 women (mean age: 56.07 years; SD: 8.88 years) were included in this study. Demographic characteristics of the women are presented in Table 1, which shows the baseline characteristics of the study population stratified by AAM. The mean age of menarche was 15.83 (SD of 1.89) years. Because of the small group numbers for each subject having earlier ages of menarche, we combined subjects aged 11 and 12 years of menarche. At baseline, women with earlier menarche were slightly younger, had higher BMI and higher alcohol consumption, and more frequently lived in urban areas (Table 1). The individuals with early menarche were more likely higher educated and had higher monthly incomes. However, we observed no association of OBS scores as an oxidative stress-related exposure and CRP levels as an inflammation marker with AAM. All women with early menarche had never regularly smoked.

### 3.2. Risks of Metabolic Syndrome and Its Association with Triglyceride by Age at Menarche

Figure 1 shows the ORs of MetS by each year of AAM (from 12 to 17 years). Compared with the reference group (age at 16 years), the risk of incident MetS was significantly increased only at ≤12 years of AAM with 1.91 (95% CI, 1.03–3.55, *P* < 0.05), after adjustment for area, age, income, education, and CRP levels. We also investigated ORs between AAM and five individual components of MetS. Only hypertriglyceridemia was significantly increased at ≤12 years of AAM with 1.99 (95% CI, 1.16–3.41, *P* < 0.01, Figure 2). However, other phenotypes did not significantly associate with AAM (Supplemental Figure S1).

We then examined the association between AAM and each component of MetS, separately. Linear regression analysis (Table 2) showed that women with earlier AAM had higher waist circumference and TG, compared with those with average AAM, after adjusting for the same covariates (*P* < 0.05). The corresponding regression coefficients of waist circumference and TG were 3.54 (95% CI = 1.53–5.55, *P* = 0.01) and 37.83 (95% CI = 16.00–59.66, *P* = 0.02), respectively. From these results (Figure 2 and Table 2), early menarche (12 years and before) is associated with increased risk of MetS, and this association appeared to be mediated mainly by increased TG of MetS.

### 3.3. Interaction between Genotypes and Age at Menarche on Triglyceride

As we considered that AAM was a modulator for the development of MetS, we conducted the joint analysis of the main SNP effect and its interaction effect with AAM interaction on MetS, and its individual traits, for all 352,228 SNPs. A list of SNPs associated with AAM-TG interactions, at *P* < 7 × 10^−7^, by joint test, is provided in Table 3. The strongest statistical evidence for SNP × AAM joint interaction was an SNP of rs6589566, located on the chromosome 11, in zinc finger protein 1 (ZPR1) (*P* = 9.54 × 10^−7^). We found that main genetic effects of SNPs located chromosomes 11 and 8 were the primary contributors to these joint associations. Specifically, we identified SNP rs1501675, located within an intronic region of *ARHGEF28*, as the most significant SNP, with *P* = 6.12 × 10^−8^ for AAM-to-TG. Other interesting SNPs were located near (±2 kb) or within the loci *LOC10537172*, *SCG2*, *TTT4*, and *LOC107986425* (all nominal *P* values < 5 × 10^−4^).

Based on these findings, we tested pathway level-based interaction between AAM and genetic variation, according to SNPs with significant *P* values for enriched biological processes, in TG. When mapping SNPs were limited to 20 kb regions flanking a gene, three pathways, type 2 diabetes mellitus, stress-activated protein kinase (SAPK) signaling pathway, and Jun amino-terminal kinases (JNK) cascade, were significantly enriched, with association signals and FDR < 0.05 (Table 4).

## 4. Discussion

In this study, we observed that early menarche (at or before 12 years of age) was associated with high prevalences of MetS; however, later AAM showed no significant association. In particular, this trend appeared to be mediated by increased TG, with earliest menarche ages associated with increased TG, in our study population. A number of studies have previously investigated the association between menarcheal age and MetS with elevated blood glucose or BMI or TG [30–32]. Recently, two studies have examined the association between AAM and risk of MetS in the Korean population. One study, of 1464 Korean women, showed that the relative risk of MetS was 3.84 (96% CI: 1.52–9.70) for menarche at age <12 years in premenopausal women (from the KNHANES database) from 2007 to 2009, along with higher blood pressure [33]. Won et al. also recently reported that 12,336 participants with early menarche (age < 12 years) were at increased risk of MetS (OR = 1.35, 95% CI: 1.03–2.12), with higher prevalence of hypertension and diabetes in KNHANES from 2010 to 2013 [19].

Puberty is the transition to adulthood that culminates in the production of mature gametes and the initiation of reproductive activity. The process begins within the central nervous system, where gonadotropin-releasing hormone (GnRH) neurons are activated to release the neurohormone, stimulating pituitary gonadotropic hormone secretions that in turn direct gonadal steroid hormone production. Therefore, a younger AAM is associated with higher cumulative exposure to ovarian hormones [34]. Estrogen increases TG by promoting synthesis of TG in the liver and secreting TG into the circulation as very-low-density lipoprotein (VLDL) particles [35]. TG levels correlatively increase during pregnancy with estrogen-induced stimulated secretion of hepatic TG-rich lipoprotein [36]. However, the etiology of MetS, with regard to its association with TG, has yet to be unraveled completely with consideration of AAM.

Several studies have also demonstrated the importance of environmental triggers (including endogenous and exogenous exposure to hormone) in the development of chronic disease. Therefore, assay of gene-environmental interactions helps to understand the etiology of disease. Although several GWA studies have investigated AAM and breast cancer risk [4, 37], few GWA studies have focused on AAM and MetS with its components. Therefore, we conducted pathway analysis for investigating interaction of genetic variants and TG using GSEA. We found that T2 diabetes mellitus, stress-activated protein kinase, and JNK cascade pathways were associated with AAM on TG levels.

During puberty, there are proinflammatory and prooxidative changes and relative insulin resistance, which also play a role in the development of T2D [38, 39]. Early puberty may be causally related to lower insulin sensitivity and inflammatory changes [40]. Reductions of insulin sensitivity and compensatory hyperinsulinemia are physiological during puberty, and this partly reflects the effects of increased growth hormone and IGF-1. Recent study reported that GnRH signaling may regulate T2D using pathway enrichment analysis [41]. However, it was reported that obesity drives metabolic risk in the prepubertal population rather than premature adrenarche [42]. More detailed mechanisms of the synergistic effects of early menarche and TG require further investigation.

SAPK/JNK are members of the MAPK family and are activated by a variety of environmental stresses, inflammatory cytokines, and growth factors. GnRH can activate the JNK/SAPK, p38, and ERK5 cascades in different cell models with varying kinetics [43]. In addition, JNKs may be a central mediator of impaired glucose metabolism and insulin resistance [44]. JNKs are also activated in obesity in numerous metabolically important cells and tissues such as adipose tissue, macrophages, the liver, skeletal muscle, and regions of the brain and pituitary. Recent studies have clearly established the important roles JNK signaling fulfils in macrophages, the liver, and cells of the anterior pituitary. Macrophage TG accumulation upregulates PON2 expression via the MEK/JNK/c-Jun pathway, and these effects could be related, at least in part, to cellular TG-induced ROS formation [45]. Collectively, these studies place JNKs as important mediator of disruptions to metabolic homeostasis. In addition, 2-methoxyestradio activated SAPK/JNK in endothelial cells in a concentration-dependent manner [46]. Although several studies shed light on the associations of stress-related pathway and TG, assessing interaction of AAM is still challenging.

Our study provides some interesting results on the interplay between AAM and the genome for TG; however, some limitations need to be considered. One of them is the relatively small sample size of women with early menarche, which may provide a lack of statistical power, limiting the magnitude of interaction effects. In addition, another weakness could be the cross-sectional design of the study, with self-reported medical histories and recalled AAM. Even though AAM can be recalled by women with moderate accuracy, the use of self-reported menarcheal age may lead to some degree of recall bias [47], and recall can be influenced by the individual's current health condition [48]. Since TG might be associated with other chronic diseases such as cardiovascular disease or hyperlipidemia [49], we tried to investigate the association between TG and other chronic diseases such as hyperlipidemia and coronary artery disease (CAD) in the study participants. Based on self-reported data to chronic diseases, we could not investigate the association between TG and other chronic diseases such as hyperlipidemia and CAD due to this small sample size lack of information. Considering confounding factors on TG, we could not investigate the effect of lipid-lowering drug and estrogen use on TG due to the small size. Due to lack of relevant information, the confounding factors mentioned above are excluded from our analysis, which may limit the strength of our findings.

This is a first attempt of a genome-wide gene-AAM interaction study on MetS components in Korean women. We identified that type 2 diabetes mellitus, the SAPK signaling pathway, and JNK cascade were associated with TG including the genetic interaction with AAM. These findings may help us understand the role of AAM on the development of MetS and gene-environment interactions that confer MetS susceptibility.

## Figures and Tables

**Table 1:** Characteristics of study subjects.

	Age at menarche (years)						All	*P* ^b^
	≤12	13	14	15	16	≥17		
Women, *n* (%)	77 (2.4)	269 (8.5)	531 (16.6)	669 (21.1)	689 (21.7)	945 (29.8)	3180	
Age at enrolment (years)	51.25 ± 6.92	52.58 ± 7.74	53.33 ± 8.47	54.92 ± 8.80	56.94 ± 8.82	59.18 ± 8.49	56.07 ± 8.88	<0.001
Area, *n* (%)								
Rural (Ansung)	26 (34)	83 (31)	199 (37)	339 (51)	412 (60)	640 (68)	1699	<0.001
Urban (Ansan)	51 (66)	186 (69)	332 (63)	330 (49)	277 (40)	305 (32)	1481	
BMI (kg/m^2^)	25.55 ± 3.37	24.62 ± 3.42	24.78 ± 3.07	24.72 ± 3.20	24.86 ± 3.16	24.89 ± 3.12	24.80 ± 3.16	0.848
Current smokers, *n* (%)	0 (0)	3 (1.1)	9 (1.7)	11 (1.6)	9 (1.3)	27 (2.9)	59 (1.8)	0.177
Alcohol consumption (g/l)	3.52 ± 11.18	1.51 ± 5.36	1.49 ± 5.28	1.40 ± 6.85	1.41 ± 6.16	1.28 ± 4.96	1.44 ± 5.96	0.068
Age at menopause	48.26 ± 3.83	47.52 ± 5.61	47.60 ± 4.62	47.87 ± 5.40	47.83 ± 5.11	47.90 ± 5.24	47.82 ± 5.16	0.718
OBS score	9.34 ± 2.41	9.39 ± 2.43	9.53 ± 2.56	9.19 ± 2.47	8.85 ± 2.53	8.91 ± 2.50	9.10 ± 2.51	0.698
CRP (mg/dl)	1.04 ± 1.34	1.22 ± 1.10	1.33 ± 1.24	1.35 ± 1.23	1.40 ± 1.27	1.53 ± 1.44	1.41 ± 1.30	0.07
Education, *n* (%)								<0.001
Less than elementary school	7 (9.1)	20 (7.4)	55 (10.4)	95 (14.2)	138 (20.1)	225 (23.8)	540 (17)	
Middle school graduate	24 (31.2)	111 (41.3)	185 (35)	305 (45.6)	384 (55.8)	575 (60.8)	1584 (50)	
High school and above	46 (59.7)	138 (51.3)	289 (54.6)	269 (40.2)	166 (24.1)	141 (24.5)	1049 (33)	
Income, *n* (%)								<0.001
<100^a^	16 (20.8)	61 (22.7)	138 (26.4)	243 (36.6)	305 (44.8)	502 (53.1)	1265 (40.2)	
100–200	23 (29.9)	59 (21.9)	112 (21.5)	144 (21.7)	156 (22.9)	220 (23.3)	714 (22.7)	
≥200	38 (49.4)	149 (55.4)	272 (52.1)	277 (41.7)	220 (32.3)	214 (22.6)	1170 (37.1)	

Values are expressed as means ± SDs (standard deviations) or number (%). BMI: body mass index; OBS: oxidative balance score; CRP: C-reactive protein; ^a^10^4^ KRW: equivalent with 1000 US dollar in 2014. ^b^ *P* value was examined by the Kruskal-Wallis test or chi-square test.

**Table 2:** Association of age at menarche with metabolic traits (data are expressed as beta coefficients, with 95% confidence intervals, for linear regression adjusted for age, area, education, income, and CRP level) in KARE.

Metabolic traits	Age at menarche (years)						*P* ^a^
	≤12	13	14	15	16	≥17	
	Beta coefficient (95% CI)						
SBP (mmHg)	2.22 (−1.65–6.10)	−1.51 (−3.82–0.80)	1.17 (−0.64–2.99)	0.54 (−1.11–2.18)	0 (−1.66–1.66)	−0.004 (−1.53–1.52)	0.27
DBP (mmHg)	1.26 (−0.98–3.50)	−0.34 (−1.71–1.03)	0.44 (−0.63–1.51)	0.42 (−0.56–1.39)	0 (−0.96–0.96)	0.58 (−0.32–1.49)	0.44
FI (mg/dl)	−0.02 (−0.92–0.88)	0.07 (−0.51–0.65)	−0.002 (−0.47–0.46)	−0.24 (−0.64–0.17)	0 (−0.40–0.40)	−0.40 (−0.77–0.03)	0.91
FG (*μ*U/ml)	2.19 (−1.13–5.51)	−0.50 (−2.42–1.42)	0.71 (−0.94–2.37)	0.63 (−0.94–2.19)	0 (−1.44–1.44)	0.97 (−0.58–2.5)	0.12
WC (cm)	3.54 ^∗^ (1.53–5.55)	0.79 (−0.42–2.00)	0.55 (−0.40–1.49)	0.26 (−0.63–1.15)	0 (−0.86–0.86)	−0.52 (−1.33–0.29)	<0.01
TG (mg/dl)	37.83^∗^ (16.00–59.66)	4.67 (−6.93–16.26)	3.15 (−6.60–12.90)	1.80 (−6.29–9.90)	0 (−8.24–8.24)	−0.004 (−7.81–7.80)	0.02
HDL (mg/dl)	−0.07 (−2.48–2.33)	0.52 (−0.94–1.99)	0.81 (−0.38–2.00)	0.11 (−0.95–1.17)	0 (−1.03–1.03)	0.66 (−0.31–1.64)	0.71

SBP: systolic blood pressure; DBP: diastolic blood pressure; FI: fasting insulin; FG: fasting glucose; WC: waist circumference; TG: triglyceride; HDL: high-density lipoprotein. ^a^Age at menarche as a dummy variable (≤12 years: 0, >12 years: 1), ^∗^ *P* < 0.01.

**Table 3:** Discovery GWAS top hits for joint and 1df interaction of gene age at menarche on triglyceride.

SNP ID	Nearest gene	Chr	Ref/var	Position	MAF	Test of interaction	
						*P*_joint_	*P*_int_
rs6589566	*ZPR1*	11	A/C	116,781,707	0.22	2.92 × 10^−12^	0.15
rs10503669	*LPL*	8	A/C	19,990,179	0.12	3.87 × 10^−9^	0.03
rs603446	*ZPR1*	11	C/T	116,783,719	0.23	4.82 × 10^−9^	0.67
rs17482753	*LPL*	8	G/T	19,975,135	0.13	1.40 × 10^−8^	0.02
rs17410962	*LPL*	8	A/G	19,990,569	0.13	1.48 × 10^−8^	0.02
rs11216186	*SIK3*	11	C/T	116,913,976	0.13	2.41 × 10^−8^	0.03
rs17120157	*SIK3*	11	C/T	116,941,912	0.13	6.43 × 10^−8^	0.03
rs10892068	*SIK3*	11	C/T	117,057,830	0.13	7.34 × 10^−8^	0.03
rs17120293	*SIK3*	11	C/T	117,074,130	0.13	7.44 × 10^−8^	0.03
rs12292858	*SIK3*	11	A/C	116,943,263	0.18	9.76 × 10^−8^	0.09
rs2044426	*SIK3*	11	C/T	116,885,467	0.10	1.52 × 10^−7^	0.02
rs6073350	*JPH2*	20	A/T	44,145,104	0.04	1.61 × 10^−7^	7.14
rs12279433	*SIK3*	11	G/T	116,877,505	0.10	1.69 × 10^−7^	0.02
rs1501675	*ARHGEF28*	5	A/C	73,695,382	0.02	1.77 × 10^−7^	6.12 × 10^−8^
rs1014492	*SCG2*	2	A/G	223,633,665	0.10	2.23 × 10^−7^	4.49 × 10^−4^
rs11216315	*PCSK7*	11	C/G	117,209,924	0.13	2.94 × 10^−7^	0.04
rs11216126	*BUD13*	11	A/C	116,746,524	0.20	3.60 × 10^−7^	0.12
rs7513082	*LOC10537172 (UBQLN4)*	1	A/C	156,031,555	0.02	4.01 × 10^−7^	5.82 × 10^−8^
rs13046	*TTT4*	1	A/C	54,742,081	0.01	6.18 × 10^−7^	1.83 × 10^−5^
rs16875865	*LOC107986425 (LHFPL2)*	5	A/G	78,746,592	0.01	6.40 × 10^−7^	4.60 × 10^−7^

**Table 4:** Pathway-based analysis of interaction between early menarche and genetic variation for triglyceride in Korean women.

Pathways	Description	FDR	Significant genes	Selected genes	All genes
Type 2 diabetes mellitus	KEGG type 2 diabetes mellitus	0.016	17	33	44
Stress-activated protein kinase signaling pathway (GO: 0031098)	Stress-activated protein kinase (SAPK) cascade	0.019	14	38	49
JNK cascade (GO: 0007254)	A cascade of protein kinase activities, culminating in the phosphorylation and activation of a member of the JUN kinase subfamily of stress-activated protein kinases	0.021	14	37	47

## Data Availability

GWAS dataset and epidemiological data for KARE project are third party data and are available under the approval of the data access committee of the National Biobank of Korea, who can be contacted at biobank@korea.kr.

## Supplementary Materials

Supplementary Materials Supplemental Figure S1. Risk of components of MetS in each age of menarche. ORs are adjusted by area, age, income, education, and C-reactive protein levels. Reference category is menarche at 16 years of age. Size of rectangle represents number of samples. A: high blood pressure; B: high fasting glucose; C: abdominal obesity; D: low HDL cholesterol. Supplemental Figure S2. Quantile-quantile plot of observed *P* values vs. expected *P* values (on a −log10 scale) for joint and interaction analyses of SNP and age at menarche on TG. A: interaction test; B: joint test; C: interaction test by reverse normal distribution; D: joint test by reverse normal distribution.
