# Supplementary material for: Effect of Interaction between Early Menarche and Genetic Polymorphisms on Triglyceride
Source: Oxid Med Cell Longev. 2019 Feb 25;2019:9148920. doi: 10.1155/2019/9148920 (PMC6410422; doi:10.1155/2019/9148920)
Supplement: Supplementary Materials — Supplemental Figure S1. Risk of components of MetS in each age of menarche. ORs are adjusted by area, age, income, education, and C-reactive protein levels. Reference category is menarche at 16 years of age. Size of rectangle represents number of samples. A: high blood pressure; B: high fasting glucose; C: abdominal obesity; D: low HDL cholesterol. Supplemental Figure S2. Quantile-quantile plot of observed P values vs. expected P values (on a −log10 scale) for joint and interaction analyses of SNP and age at menarche on TG. A: interaction test; B: joint test; C: interaction test by reverse normal distribution; D: joint test by reverse normal distribution. [file 9148920.f1.doc]

SUPPLEMENTAL INFORMATION:

**Effect on the interaction between early menarche and genetic polymorphisms on triglyceride**

Ho-Sun Lee, Sangseob Leem and Taesung Park^*^

Interdisciplinary Program in Bioinformatics and Department of Statistics, Seoul National University, 1 Kwanak-Ro, Kwanak-gu, Seoul, 151-747, Republic of Korea.

**Supplemental Figure S1. Risk of components of MetS in each age of menarche**. ORs are adjusted by area, age, income, education, and C-reactive protein levels. Reference category is menarche at 16 years of age. Size of rectangle represents number of samples. A, high blood pressure; B, high fasting glucose; C, Abdominal obesity; D, low HDL cholesterol


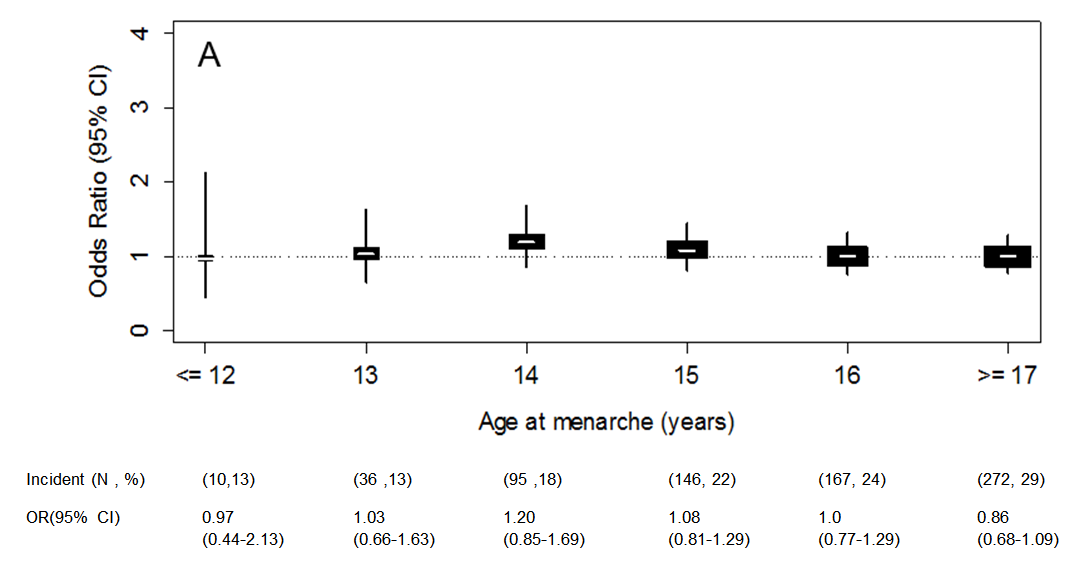


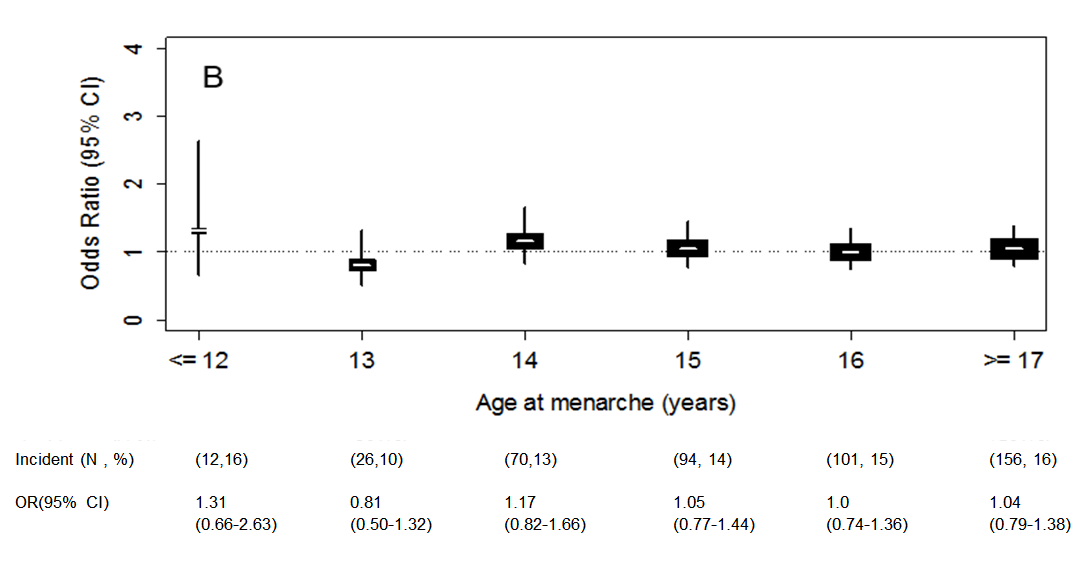


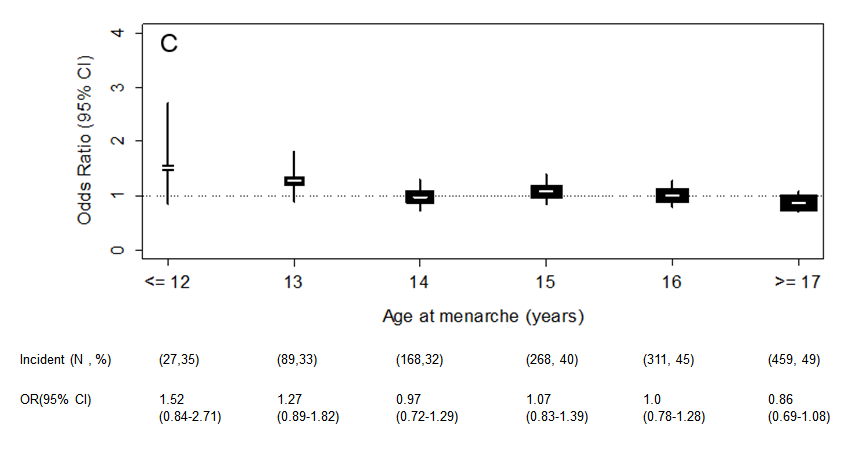


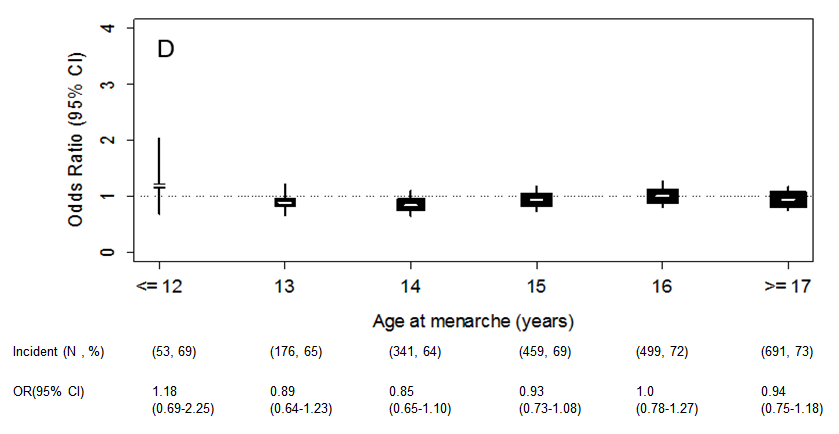


**Supplemental Figure S2.** Quantile-Quantile Plot of Observed P values vs Expected P values (on a –log10 Scale) for joint and interaction analyses of SNP and age at menarche on TG. A, interaction test; B, Joint test; C, interaction test by reverse normal distribution; D, Joint test by reverse normal distribution

**
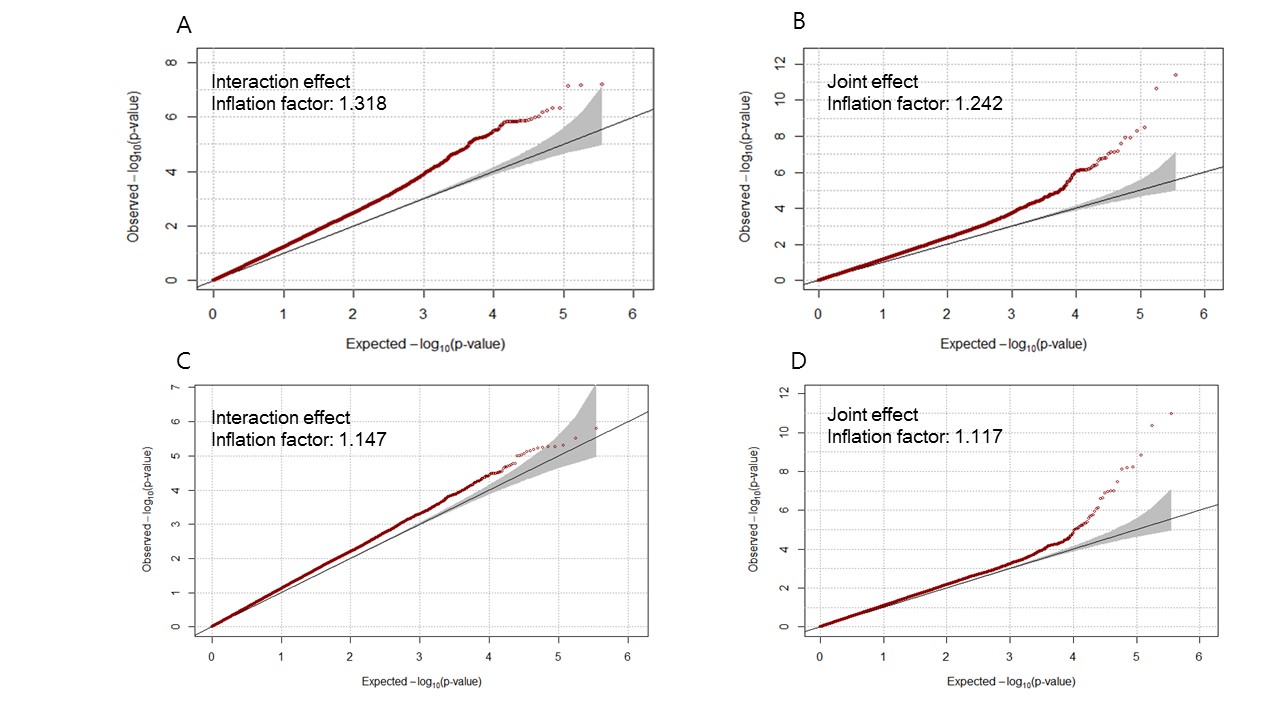
**
